# Impact of water models on the structure and dynamics of enzyme tunnels

**DOI:** 10.1016/j.csbj.2024.10.051

**Published:** 2024-11-02

**Authors:** Aaftaab Sethi, Nikhil Agrawal, Jan Brezovsky

**Affiliations:** a Laboratory of Biomolecular Interactions and Transport, Department of Gene Expression, Institute of Molecular Biology and Biotechnology, Faculty of Biology, Adam Mickiewicz University, Uniwersytetu Poznanskiego 6, Poznan 61–614, Poland; b Latvian Institute of Organic Synthesis, Aizkraukles 21, LV, Riga 1006, Latvia; c International Institute of Molecular and Cell Biology in Warsaw, Ks Trojdena 4, Warsaw 02–109, Poland

**Keywords:** Channels, Enzymes, Explicit water models, Ligand transport, Molecular dynamics simulations, Tunnels

## Abstract

Protein hydration plays a vital role in many biological functions, and molecular dynamics simulations are frequently used to study it. However, the accuracy of these simulations is often sensitive to the water model used, a phenomenon particularly evident in intrinsically disordered proteins. Here, we investigated the extent to which the choice of water model alters the behavior of complex networks of tunnels within proteins. Tunnels are essential because they allow the exchange of substrates and products between buried enzyme active sites and the bulk solvent, directly affecting enzyme efficiency and selectivity, making the study of tunnels crucial for a holistic understanding of enzyme function at the molecular level. By performing simulations of haloalkane dehalogenase LinB and its two variants with engineered tunnels using TIP3P and OPC models, we investigated their effects on the overall tunnel topology. We also analyzed the properties of the primary tunnels, including their conformation, bottleneck dimensions, sampling efficiency, and the duration of tunnel openings. Our data demonstrate that all three proteins exhibited similar conformational behavior in both models but differed in the geometrical characteristics of their auxiliary tunnels, consistent with experimental observations. Interestingly, the results indicate that the stability of the open tunnels might be sensitive to the water model used. Because TIP3P can provide comparable data on the overall tunnel network, it is a valid choice when computational resources are limited or compatibility issues impede the use of OPC. However, OPC seems preferable for calculations requiring an accurate description of transport kinetics.

## Graphical Abstract

**Figure:**
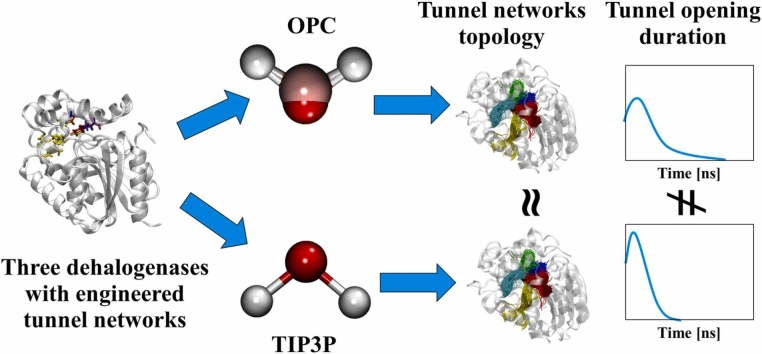

## 1 Introduction

Water is indispensable in determining the structure, stability, dynamics, and function of proteins. [1] It plays a critical role in various biological processes, such as protein folding and enzymatic catalysis. [2], [3] Interfacial/bound and internal/buried water molecules can affect protein-protein association, protein-ligand interactions, [4], [5] protein thermal stability, and peptide/ligand binding affinity. [6], [7] Furthermore, proteins also perturb the dynamics of water molecules. The surface of proteins is highly heterogeneous due to geometrical disorder and the differing energetics of the local hydrogen bonds between water and protein sites. [8], [9] This heterogeneity can lead to increased residence times of some water molecules in the vicinity of protein surfaces. It has been estimated that the residence time of such dampened water molecules near the protein surface could range from nanoseconds to microseconds. [10], [11] However, the residence time of water molecules buried inside the protein structure could reach up to milliseconds. [1], [12] In addition, proximity to proteins can also affect the reorientational and translational behavior of water molecules, with NMR studies observing that water reorientation and translation near globular proteins is 3 to 5 times slower than in bulk. [9], [13], [14].

All-atom molecular dynamics (MD) simulations have been widely employed to investigate protein-water interactions and have provided insight into water properties and their effects on biomolecules. [15], [16] The accuracy of simulations involving proteins immersed in explicit water molecules depends on the choice of the protein force field and the water model used. [17], [18], [19] While there are numerous water models, the most commonly applied are single point charge (SPC), [20] transferable intermolecular potential (TIP), [21] and optimal point-charge (OPC). [22] The SPC family includes three-point charges placed on the nuclei and a single 12–6 Lennard-Jones term centered on the oxygen or hydrogen atoms. [20] The TIP family consists of 3-, 4-, and 5-point water models named TIP3P, TIP4P, and TIP5P, respectively. [23] Finally, the OPC family includes 3- and 4-point water models, OPC3 and OPC, respectively. [24] These water models, while imperfect, provide a good compromise between accuracy and computational efficiency. For example, the kinetic properties of TIP3P, such as self-diffusivity and viscosity, do not agree well with experimental observations, but its thermodynamic properties are considered a reasonable approximation. [1], [25] Historically, most of these water models have been developed in conjunction with specific protein force fields, such as SPC with GROMOS, [26] TIP3P with AMBER and CHARMM, [27], [28] and TIP4P with OPLS force fields. [29] Due to its less stringent coupling to particular water models, AMBER force fields have been shown to work effectively with TIP4P, TIP5P, and OPC water models. [30], [31].

To date, many studies have investigated discrepancies arising from the use of different explicit water models. For example, Anandakrishnan *et al.* used TIP3P and TIP4P/Ew models to investigate the protein folding landscape of the mini-protein CLN025 and found nearly order-of-magnitude differences in the models’ abilities to predict the fraction of unfolded states. The origin of this discrepancy was traced to water-water electrostatic interactions. [19] Gupta *et al.* studied the temperature-dependent glass transition of the Trp-cage mini-protein using mTIP3P, TIP4P, and TIP4PEw models and found that the TIP3P model resulted in a decreased number of water molecules packed in the first hydration shell of the Trp-cage surface. [32] Importantly, conformational ensembles of intrinsically disordered proteins simulated with different water models were shown to differ, often being too compact compared to various experimental measurements, which could primarily be attributed to weaker dispersion interactions. [33], [34], [35], [36], [37] Considering the impact of models on the binding of water molecules to proteins, Fadda *et al.* performed simulations of the Concanavalin A protein in both free and ligand-bound forms using TIP3P, TIP4P, and TIP5P models. The authors demonstrated that the binding energies of isolated water molecules are highly sensitive to the model used. [38] Finally, a study on water diffusion through aquaporin AQP1 by Gonzalez *et al*. compared TIP3P, OPC, and TIP4P/2005 models. [39] They showed that OPC and TIP4P/2005 were able to reproduce protein-water interactions in good agreement with experimental data, while the application of the TIP3P model resulted in an overestimation of the diffusibility of water molecules. A similar observation was recently made by Thirunavukarasu *et al*. for water transport via tunnels in three distinct globular enzymes. [40] Overall, these studies suggest that the accuracy of simulations is sensitive to the water models used, which were initially developed with relatively stable globular proteins in mind. This is particularly relevant when considering less ordered protein fragments and the interactions and behavior of water molecules buried within the protein.

From this perspective, it is pertinent to consider another structural element of proteins: tunnels. These empty spaces connect the surrounding solvent to the buried functional site within certain enzymes. [41], [42], [43] Tunnels are often equipped with molecular gates that switch between closed and open states, making them transient. [44] The dynamics and structure of tunnels play a critical role in the entry of substrates into the active site and the release of products into the bulk solvent. [44], [45] Another important role of tunnels is to act as selective filters, allowing only cognate molecules of substrates or solvents to enter the active site. [46], [47], [48] Interestingly, the sensitivity of tunnels to solvent composition was demonstrated by Stepankova *et al.*, who showed significant alterations in the tunnel entrances of three different dehalogenases when exposed to three different aqueous solutions containing water-miscible organic co-solvents. [49] Given that tunnels are critical for enzyme function, can be rationally engineered to provide improved biocatalysts, [41], [45] and represent attractive drug targets, [50] it is essential to understand the role of water models in shaping the tunnel networks observed in the simulated conformational ensembles.

**Fig. 1:**
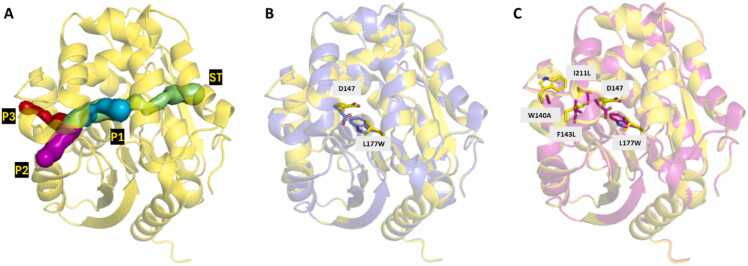
**: Illustration of the tunnel networks analyzed in the study and residue differences between the three systems**. **A**) The P1 (cyan), P2 (magenta), P3 (red), and ST (green) tunnels in the structure of LinBWT (PDB ID: 1MJ5), depicted in yellow. **B**) The L177W mutation in LinB32 (PDB ID: 4WDQ) is shown in purple, aligned with LinBWT (yellow). This mutation results in the closure of the P1 tunnel in LinB32 when a hydrogen bond forms between W177 and D147. **C**) The four mutations (L177W, W140A, F143L, and I211L) present in LinB86 (PDB ID: 5LKA) are shown in magenta, aligned with LinBWT (yellow). The three mutations, excluding L177W, are responsible for activating the P3 tunnel.

In the present study, we investigated the effect of two different water models on the structure and dynamics of complex tunnel networks in enzymes. For this purpose, we chose the TIP3P water model because of its widespread use, [51] and the OPC model, which is known for effectively reproducing bulk water properties. [24] As a model system with well-understood and diverse tunnel networks, we used members of the haloalkane dehalogenase (HLD) family, LinBWT and its two engineered variants, a single-point mutant LinB32 and a four-point mutant LinB86 **(**Fig. 1**)**. [45], [52], [53] Structurally, these enzymes belong to the α/β-hydrolase superfamily, characterized by their main and cap domains, with the buried active site at their interface. [54] HLDs have a long-standing history as a model system for studying the catalytic action of enzymes with buried active sites due to the wealth of data collected on the transport components of their function. [55] This includes identifying rate-determining steps, [45], [52], [53] numerous crystal structures, and in-depth studies of the effects of mutations in the tunnel networks of these enzymes. [41] The three HLD model systems used here have different networks of primary and auxiliary tunnels. In LinBWT, the main tunnel (P1) is prominently open, but in the LinB32 and LinB86 variants, the P1 tunnel is blocked by the L177W mutation, hindering substrate and product transport. [45], [53] In addition, Trp177 forms a hydrogen bond with Asp147, which sets up the cap domain gate of the P1 tunnel. [52] In LinB86, three additional mutations (W140A, F143L, and I211L) were introduced into the auxiliary access tunnel P3, resulting in the activation of the P3 tunnel and enhanced flexibility of the cap domain gate of the P1 tunnel. [45], [52] These different tunnel networks allowed us to analyze the extent to which the water model used affects our ability to detect relevant tunnels and the duration for which the tunnels remain open. Additionally, we analyzed how quickly the tunnels were detected, the number of tunnels detected, and probed their bottleneck radius (BR), examining any significant differences between the two models.

## 2 Methods

To study the effect of different water models on tunnel dynamics, we performed simulations for LinBWT, LinB32, and LinB86 enzymes using TIP3P and OPC water models. The initial structures of LinBWT (PDB ID: 1MJ5), [56] LinB32 (PDB ID: 4WDQ), [45] and LinB86 (PDB ID: 5LKA) [45] were prepared for simulations as previously described. [45] Water molecules were initially placed around the protein structures in three steps. In the first step, 3D-RISM theory was used to add initial water molecules to the protein structures according to the Placevent algorithm. [57], [58] In the second step, the predicted water molecules were merged with crystallographic water molecules. In the last step, water molecules with oxygen atoms within 2 Å (1 Å = 0.1 nm) of protein atoms were removed. Truncated octahedral water boxes (TIP3P and OPC) were added to a distance of 10 Å from any atom in the respective systems, and ions (Na^+^ and Cl^-^) were added to achieve a final concentration of 0.1 M using the *tleap* module of AMBER 18. [59] Hydrogen mass repartitioning (HMR) was performed using the *parmed* module of AMBER 18 to enable a 4 fs timestep. [60] Here, we would like to acknowledge that the implementation of HMR may impact the time scales of the studied processes, as evidenced in recent literature. [61], [62] Consequently, in investigations aimed at determining absolute transport rates, as opposed to the primarily comparative analyses conducted in this study, it might be advisable to refrain from employing HMR.

The system energy was minimized using the *pmemd* module of AMBER 18, by using 100 of the steepest descent steps followed by 400 conjugate gradient steps within five rounds of decreasing harmonic restraint as follows: 500 kcal·mol^−1^·Å^−2^ applied to all heavy atoms of the protein, and then 500, 125, 25, and 0 kcal·mol^−1^·Å^−2^ on backbone atoms only. The equilibration of the systems was performed in four stages. In the first stage, 20 ps NVT simulations were performed to gradually heat the systems from 0 K to 200 K, followed by 1 ns NVT simulations to reach the target temperature of 310 K. In these two stages, harmonic restraints of 5.0 kcal·mol^−1^·Å^−2^ were applied to the heavy atoms of the proteins, and the temperature was controlled using a Langevin thermostat, [63] with a collision frequency of 2.0 ps^−1^. The third stage consisted of 1 ns NPT simulations performed at 310 K using the Langevin thermostat at a constant pressure of 1.0 bar, with a Monte Carlo barostat while keeping restraints of 5.0 kcal·mol^−1^·Å^−2^ on backbone atoms only. The final stage employed the same settings as the third but without any positional restraints.

Finally, unrestrained NPT simulations were run for all investigated systems for 500 ns, with data saved to trajectories at 20 ps intervals. All simulations employed the SHAKE algorithm [64] to fix bonds containing hydrogens and used periodic boundary conditions with a nonbonded cutoff of 8 Å. The simulations were performed using the *pmemd.cuda* module of AMBER 18 with the ff14SB force field. [65] Three replicated simulations were conducted for LinBWT in TIP3P and OPC water models. In contrast, more simulations were performed to obtain four replicates (two open and two closed forms) of LinB32 and LinB86 in both water models **(**Table S1**)**. The initial 100 ns of simulations were dedicated to water and structural equilibration, leaving 400 ns (20,000 frames) of production simulations for further analyses per replicate.

The dynamic behavior of tunnels was analyzed using a divide-and-conquer approach, [66] utilizing CAVER 3.0. [67] Briefly, 20,000 pdb files were generated from each run, corresponding to 20,000 frames. These were divided into 10 batches of 2000 pdb files each. The Caver configuration file was prepared, defining the starting point by the center of mass of Trp109, His272, and Asn38. Tunnels were investigated in each trajectory snapshot using a probe radius of 0.7 Å, shell radius of 3 Å, shell depth of 4 Å, and a clustering threshold of 4.5 Å. A filtering criterion was applied to the Caver run, retaining only those tunnels observed in more than 5 % of the frames. Subsequently, TransportTools was utilized to merge the tunnels. [66], [68] The Ward clustering method was applied to cluster the tunnels with a clustering cutoff of 1 Å. The minimum tunnel radius for clustering was set to 0.7 Å, and the minimum tunnel length to 5 Å. Further, the clusters generated by TransportTools were converted to Caver output format using a Python script (tt_convert_to_caver.py). [66] Finally, a comparative analysis was performed using TransportTools to generate individual supercluster statistics. The comparative group definition involved six systems: LinBWT OPC & TIP3P, LinB32 OPC & TIP3P, and LinB86 OPC & TIP3P. The Ward clustering method was applied again to cluster the tunnels with a cutoff of 1 Å, with the minimum tunnel radius for clustering set to 0.7 Å and the minimum tunnel length to 5 Å.

Root-mean-square deviation and radius of gyration were calculated for the backbone atoms (N, CA, C) of residues numbered 11–295, because residues 1–10 are loop residues. Residue-wise root mean square fluctuation (RMSF) was calculated using the backbone atoms (N, CA, C) of residues 1–295. The radial distribution function (RDF) was calculated for water (O, H1, H2 atoms) around Asp108 (CG atom), with a bin spacing of 0.1 Å and a maximum bin value of 15 Å. All these analyses were performed using the *cpptraj* module of AMBER 18. [69].

## 3 Results and discussion

### 3.1 Comparison of geometrical characteristics of main tunnels and their response to engineering between OPC and TIP3P

The production phases of all selected simulations exhibited a very stable structure concerning their initial conformations, as well as very consistent RMSF profiles between the two water models used **(**Figs. S1–S3**)**. Additionally, the relative solvation levels of the transport tunnels showed high concordance between the simulations in both water models based on their RDFs (Fig. S4).

Similarly, the comparison of the number of water molecules in the first (3.4 Å) and second (5 Å) solvation shells surrounding key active site residues (N37, D108 and H272) and bottleneck residues of the P1 tunnel (147, 177 and 248), which were consistently observed across all systems, revealed no statistically significant differences between simulations with TIP3P and OPC models **(**Table S2**)**, indicating that both models yield comparable solvation behavior in these regions. Overall, these analyses confirmed the absence of major global differences between the conformations of the analyzed enzymes and their overall solvation when simulated in TIP3P and OPC, allowing us to focus on the tunnel networks.

**Fig. 2:**
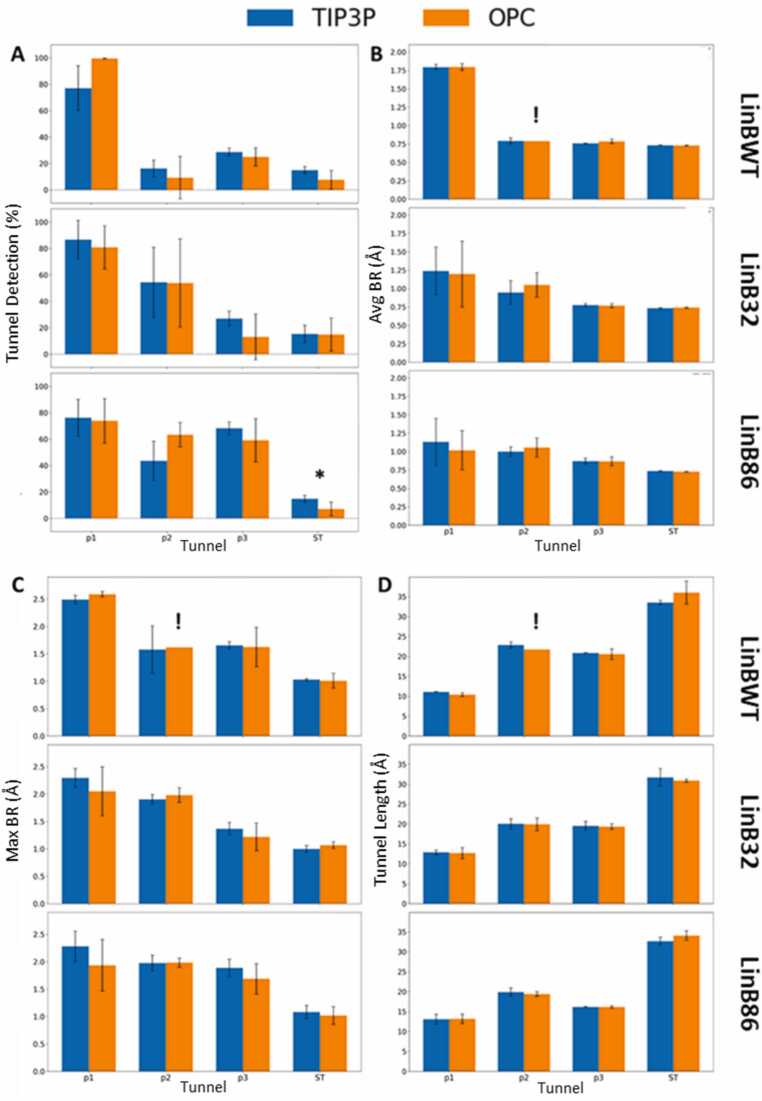
**: Frequency of tunnel detection and geometric characteristics for main and auxiliary tunnels in the three systems using TIP3P and OPC water models**. **A**) Comparison of the frequency of tunnel detection (bottleneck radium (BR) > 0.7 Å) for P1, P2, P3, and side tunnel (ST). B) Comparison of the average BR, C) the maximum BR, and D) the lengths of these four tunnels. Data represent average ± standard deviation calculated from three or four molecular dynamics (MD) simulations of LinBWT and its variants, respectively (see Table S3A). Statistically significant differences between OPC and TIP3P are indicated by asterisks above the bar plots. See also Tables S3B–D for details on the statistical tests. The exclamation mark indicates the inability to conduct statistical tests because the tunnel was found in one replicate only.

A tunnel acts as a dynamic filter that plays an essential role in enzyme activity. The opening of the tunnel facilitates the entry and exit of substrates, products, ions, and solvent molecules into and out of the enzyme. [50] To understand the effect of water models on the presence of key components of the tunnel network, we calculated how often the tunnels were identified using a spherical probe with a 0.7 Å radius. Our analysis focused on the major branches of tunnels established in the dehalogenase family, specifically P1, P2, and P3, [45], [70] as well as a recently explored side tunnel (ST). [70], [71] In addition, we compared the BR, maximum BR, and tunnel length to discern any significant differences in the structural properties of the tunnels identified by the two water models (Fig. 2 **&** Table S3A).

In LinBWT, the main tunnel (P1) could be detected for most of the simulation time (OPC: 99.65 ± 0.1 %; TIP3P: 77 ± 17 %), regardless of the water model used **(**Fig. 2**A)**, with no statistically significant differences **(**Table S3B**)**. In the simulations of LinB32 and LinB86, the effect of the L177W mutation, which hampers the opening of the P1 tunnel, was consistent between the water models (i.e., the average BR was reduced for the P1 tunnel compared to LinBWT) **(**Fig. 2**B &** Tables S3B**–D)**. Furthermore, the more frequent opening of the engineered P3 tunnel was consistently detected in LinB86 simulations with both water models **(**Fig. 2**A &** Table S3D**)**, in line with previous observations. [45] The ST exhibited the lowest frequency of occurrence in all three systems across both water models **(**Fig. 2**A)**. There was a statistically significant difference (p-value: 0.0379) in the detection of this tunnel between the simulations of LinB86 with OPC and TIP3P **(**Table S3D). Still, both models correctly ranked ST as the rarest (OPC: 7 ± 5 %; TIP3P: 15 % ± 3 %). Finally, the average lengths of P1, P2, P3, and ST were in excellent agreement between the water models **(**Fig. 2**D &** Tables S3B**–D)**. A detailed comparison of tunnel detection and geometric characteristics (average BR, maximum BR, and average length) of all tunnel clusters identified in OPC and TIP3P simulations can be found in Tables S4A**–C**. Overall, these data suggest that both water models can be used to detect similar tunnel networks with only minor differences and provide comparable insights into the effects of mutations on the tunnels.

### 3.2 Comparison of P1 bottleneck dimension between TIP3P and OPC

The architecture of enzyme access tunnels is crucial for determining ligand specificity, reaction kinetics, and overall enzyme stability. [72] Narrower tunnel bottlenecks may limit ligand transport but can reduce active site solvation and increase the probability of productive binding. [72] The residues lining these tunnels also play a significant role by lowering initial entropy and facilitating effective interactions between the ligand and the active site. [72] For instance, in the DhaA enzyme, a single point mutation (Y176A) that altered the bottleneck of the main tunnel significantly widened the tunnel, which accelerated the binding of a fluorescent probe by three orders of magnitude. [73] Due to the vital role of tunnel bottlenecks, we investigated the effect of water models on bottleneck geometry, focusing our detailed analysis on the main tunnel (i.e., P1).

**Fig. 3:**
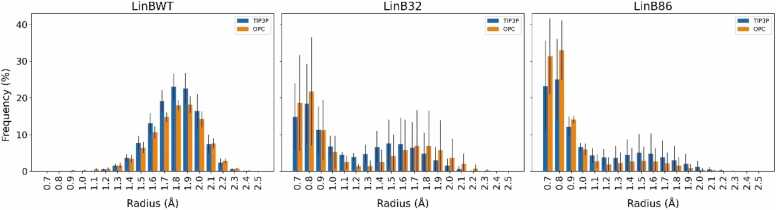
**: BR distribution of the P1 tunnel found in the LinB variants simulated in TIP3P and OPC water models**. Data represent average ± standard deviation calculated from three or four MD simulations of LinBWT and its variants, respectively. Information on the statistical significance of the differences observed between the water models is available in Table S5.

Data for tunnel geometries in LinBWT revealed that the main tunnel was qualitatively comparable in both water models, with a higher frequency observed for BR between 1.4 and 2.1 Å **(**Fig. 3**)**. Similarly, the overall trends in the frequency of tunnel geometries in LinB32 simulations were comparable between the two water models. The higher frequency distributions of BR were shifted to the lower end of the spectrum, with reasonable frequencies observed for 1.4 to 2.0 Å. For LinB86, similar profiles were seen for both water models, with very high-frequency distributions observed for shorter BR, consistent with the closing of the P1 tunnel for this variant. It is important to note that higher standard deviations were observed for LinB32 and LinB86, which can be accounted for by the inclusion of the two extremes (i.e., open and closed states). Although t-tests indicated no statistically significant differences between the water models in any of the BR ranges **(**Table S5**)**, a trend was evident: simulations with OPC often resulted in narrower tunnels with radii of 0.7 to 0.9 Å and less frequently produced open tunnels with radii ranging from 1.4 to 2.0 Å **(**Fig. 3**)**. In summary, these results indicate that the distribution of radii of the primary P1 tunnel was reasonably well maintained in simulations using either of the two water models.

### 3.3 Comparison of duration of tunnel opening between TIP3P and OPC

**Fig. 4:**
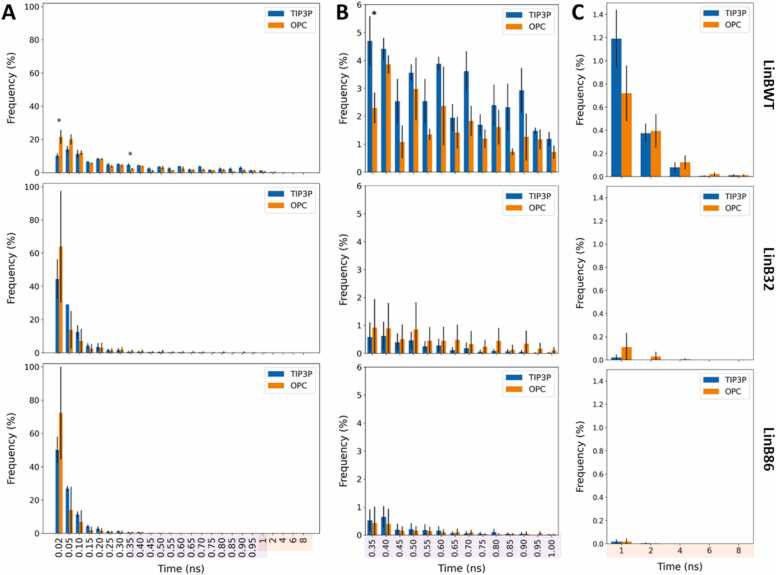
**: Duration of open states (BR ≥ 1.4 Å) of the main P1 tunnel found in the studied conformations of LinB variants simulated in TIP3P and OPC water models**. **A**) The duration of open states spans 0.02–8 ns. Data represent average ± standard deviation calculated from three or four MD simulations of LinBWT and its variants, respectively. Statistically significant differences between OPC and TIP3P are indicated by asterisks above the bar plots. See also Table S6 for details on the statistical tests. The coloring of the x-axis indicates the zoomed-on regions in panels B (violet) and C (yellow). **B**) A closer look at the duration of open states within the 0.35–8 ns range, while **C**) highlights the 1–8 ns timescale.

To understand the effect of water models on the stability of the functionally relevant open state of tunnels, we monitored the duration of the main tunnel open states (≥ 1.4 Å) before they closed again (< 1.4 Å) **(**Fig. 4**)**. Although a recent study identified a 0.7 Å radius as functionally relevant for tunnels of the α/β-hydrolase superfamily, [74] we chose a 1.4 Å threshold to specifically assess how tunnel openness influences ligand interactions. This threshold allows us to capture a broader range of tunnel dynamics, which is crucial for understanding the potential impact on ligand binding and transport. Unsurprisingly, opening events in the P1 tunnel predominantly occurred within timescales of 20 to 100 ps across all simulated systems. However, we occasionally observed openings lasting between 1 and 8 ns. Tunnel opening data for LinBWT revealed that the opening stability of the main tunnels was similar in both water models. A statistically significant difference was observed for shorter-duration tunnel openings (0.02 and 0.35 ns, p-values: 0.0240 and 0.0326), with these events being more frequent in OPC than in TIP3P **(**Fig. 4**A &** Table S6**)**. For slightly longer-duration openings, events were more frequent in TIP3P than in OPC. In LinB32, we observed a marked increase in the duration of tunnel openings in the OPC water model, reaching up to 6 ns, while being approximately 1.5 ns for TIP3P. This observation is supported by the overestimated permeability of TIP3P water molecules seen in aquaporin AQP1 [39] and the underestimated binding affinity of these molecules within the Concanavalin A protein, [38] which could diminish their contribution to stabilizing the open state. Similar to LinBWT, the differences in opening durations in LinB86 were comparable between the two models, including the stabilities of the tunnel, with the longest observed opening period ranging from 1.5 to 2.0 ns. In summary, the water models can markedly affect the stabilization of open tunnel states, which can have significant implications for their ability to transport small molecules. However, this effect appears to be system-specific. Notably, our analysis indicates a preference for long-tailed events in OPC compared to TIP3P for LinB32. Conversely, TIP3P exhibited a higher frequency of long-tailed events for LinBWT and LinB86, although these differences were not statistically significant. This suggests that the choice of water model should be made with careful consideration of the specific system under study. A consistent trend observed in all the simulated systems was the prevalence of extremely short-duration events in OPC compared to TIP3P. When evaluating trends in the three systems, the most distinct difference could be observed for the long-tailed events, wherein the frequency and duration of the prolonged open state were much greater in LinBWT when compared to LinB32 and LinB86, approximately 5 and 7 ns in TIP3P and OPC, respectively **(**Fig. 4**C)**.

### 3.4 Assessment of tunnel cluster sampling and detection rates with TIP3P and OPC

**Fig. 5:**
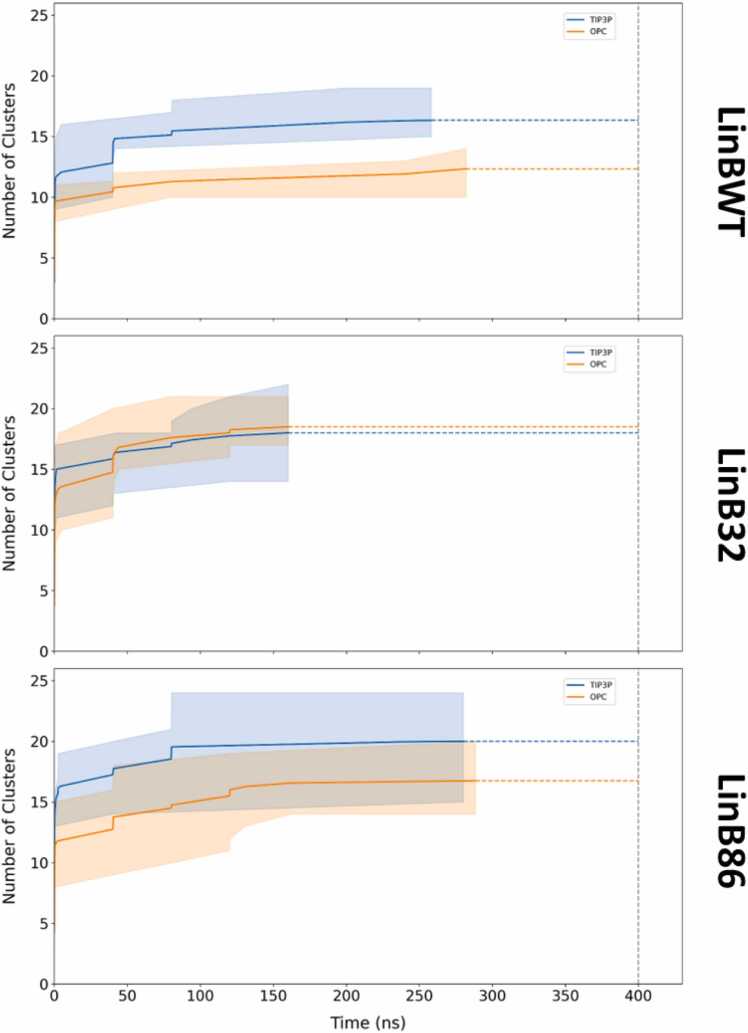
**: Average time taken to identify tunnel clusters in the studied conformations of LinB variants simulated in TIP3P and OPC water models**. The solid line represents the average number of clusters identified across three or four MD simulations of LinBWT and its variants, respectively. The shaded region represents the range of the number of clusters identified across all simulations. The vertical dotted line denotes the end of the simulation. The start of the horizontal dotted lines (blue and orange) indicates the time after which no new clusters were identified in any of the simulation replicas.

To understand the effect of water models on the detection rates and sampling of tunnel clusters, we analyzed the performance of TIP3P and OPC across the three systems. Generally, simulations using TIP3P detected more tunnel clusters and did so earlier **(**Fig. 5**)**. This difference was most pronounced in LinBWT compared to LinB32 and LinB86, indicating that the TIP3P water model rapidly induces the appearance of tunnels, potentially due to its dynamic properties. This efficiency is further evidenced in Table S1, where the molecular gate governing P1 tunnel geometry was consistently more likely to be open in TIP3P compared to OPC for LinB32 and LinB86. While TIP3P simulations resulted in the detection of a higher number of clusters within each system, these clusters tended to be the same set repeatedly. This suggests that TIP3P has a propensity for quickly identifying a consistent set of clusters. In contrast, OPC demonstrated a distinct advantage by detecting a greater variety of unique tunnel clusters across all studied systems (Tables S4A**–C)**.

Overall, OPC identified a total of 35 distinct tunnel clusters, while TIP3P detected 32 clusters. This marginal difference highlights that while TIP3P excels in speed and frequency, OPC provides a more thorough exploration, albeit at a comparatively slower pace. Notably, the unique clusters identified by OPC corresponded to tunnel cluster IDs (CIDs) 26, 27, and 36. CID 26 was identified in one simulation of LinBWT and in two simulations of LinB86; CID 27 was identified in one simulation each of LinBWT and LinB86, while CID 36 was detected in two simulations of LinB32. This distribution indicates that OPC’s ability to uncover these clusters extends across all three systems and is not merely an artifact of a particular system. Upon closer examination, all three CIDs corresponded to branches of the main tunnels. CID 26 appeared in approximately 2 % of the frames, CID 27 in about 1.5 %, and CID 36 in roughly 0.9 %. Despite their relatively low frequency of occurrence, these superclusters represent unique extensions of the main tunnel and may be particularly valuable in longer exploratory simulations, where the likelihood of observing infrequent but potentially significant features is enhanced. The detection of unique clusters by OPC could be crucial for understanding the full range of tunnel dynamics and their potential functional implications. To summarize, while TIP3P and OPC each have their strengths, the data indicates that the choice of water model should be tailored to the specific research objectives, balancing the need for rapid detection with comprehensive sampling of the tunnel networks.

## 4 Conclusion

Herein, we performed a series of MD simulations of LinBWT and its two engineered variants in TIP3P and OPC water models. Our simulations showed that the structural ensembles of these proteins behaved similarly, with both water models exploring almost identical conformations and resulting in the detection of similar networks of transport tunnels. In these simulations, the OPC model exhibited increased success in identifying narrower tunnels compared to the TIP3P simulations. However, we also observed a marked sensitivity in the stability of open states to the water model used, making the choice of model important when investigating ligand binding and unbinding kinetics, such as when estimating the residence times of inhibitors within drug discovery frameworks. [75], [76], [77].

With this in mind, future research will expand to explicitly analyze the transport of water molecules through enzyme tunnels using different models and tools such as AQUA-DUCT. [78] Finally, we would like to highlight that the 3-point TIP3P model is also an equally suitable choice as the OPC model for investigating tunnel network geometry in scenarios where available computational resources are limited (across our studied systems TIP3P achieved ∼35 % higher simulation speed than OPC) or incompatibility with a protein force field may prohibit the use of the more costly 4-point OPC model.

## Author statement

I certify that all authors have seen and approved the final version of the manuscript being submitted, that is original work by Aaftaab Sethi, Nikhil Agrawal, and Jan Brezovsky. The manuscript is not published nor under consideration for publication elsewhere.

## CRediT authorship contribution statement

**Jan Brezovsky:** Writing – review & editing, Writing – original draft, Supervision, Software, Resources, Project administration, Methodology, Funding acquisition, Data curation, Conceptualization. **Nikhil Agrawal:** Writing – original draft, Software, Methodology, Investigation, Formal analysis, Data curation, Conceptualization. **Aaftaab Sethi:** Writing – review & editing, Writing – original draft, Visualization, Validation, Software, Methodology, Investigation, Formal analysis, Data curation, Conceptualization.

## Declaration of Competing Interest

The authors declare that they have no known competing financial interests or personal relationships that could have appeared to influence the work reported in this paper.

## Data Availability

The data underlying this study are available in the published article, its supplementary information, and the Zenodo repository at https://doi.org/10.5281/zenodo.7929605. The data were deposited as plain text, PDB-formatted structural data, and AMBER-formatted MD trajectories, which can be processed using numerous freely available software packages; no tools with restricted access are needed.

## Footnotes

Appendix A Supplementary data associated with this article can be found in the online version at doi:10.1016/j.csbj.2024.10.051.
