# Supplementary material for: Impact of water models on the structure and dynamics of enzyme tunnels
Source: Comput Struct Biotechnol J. 2024 Nov 2;23:3946–54. doi: 10.1016/j.csbj.2024.10.051 (PMC11584523; doi:10.1016/j.csbj.2024.10.051)
Supplement: Supplementary file 1 — Supplementary material [file mmc1.pdf]

Supplementary Materials

Impact of water models on the structure and dynamics of enzyme tunnels

Aaftaab Sethi[1#], Nikhil Agrawal[2#], Jan Brezovsky[1,3]*

[1] Laboratory of Biomolecular Interactions and Transport, Department of Gene Expression, Institute of Molecular Biology and Biotechnology, Faculty of Biology, Adam Mickiewicz University, Uniwersytetu Poznanskiego 6, Poznan 61-614, Poland

[2] Latvian Institute of Organic Synthesis, Riga, Aizkraukles 21, LV, Riga 1006, Latvia

[3] International Institute of Molecular and Cell Biology in Warsaw, Ks Trojdena 4, Warsaw 02-109, Poland

[#] These authors contributed equally to this work.

*****Corresponding Author – Jan Brezovsky: janbre@amu.edu.pl

Text S1. The conformational state of cap domain gates in dehalogenases are structurally comparable between OPC and TIP3P.

In a previous study using both experimental and simulation techniques, Brezovsky *et al.* discovered that LinB32 and LinB86 can adopt open and closed conformations due to the marked movement of one of the cap domain helices after the hydrogen bond between the Asp147 and mutated Trp177 is broken (Brezovsky et al., 2016; Kokkonen et al., 2018). Moreover, these open and closed states were shown to be associated with very different enzymatic efficiency (Kokkonen et al., 2018). To identify the open and closed states of these proteins in our simulations, we calculated the percentage of time that the gate between residues 147 and 177 remained open for water molecules in each system (**Table S1**). Due to the lack of hydrogen bonds stabilizing the closed gate conformation, each of the three initial simulations of LinBWT with both water models exhibited an almost exclusively open state. In the case of the mutants, additional simulations had to be run to obtain at least two simulations that sampled the opened states for at least 20 % of the time. Furthermore, we found that to discover open states for LinB32 with the OPC water model required approximately twice as many screening simulations in comparison to other systems. This is consistent with a slower sampling of the opening process in OPC and the previously established enhanced structural stability of the cap-domain helical gate in this mutant (Brezovsky et al., 2016; Kokkonen et al., 2018). The final set of two simulations with the most often open state (LinB32-Open and LinB86-Open) and the two most often closed simulations (LinB32-Closed and LinB86-Closed) were assembled for each water model (**Table S1**).

Table S1. Opening of molecular gate between cap domain helices for water molecules.

Simulation Replica #	Percent of simulations with minimum distance between ASP147 and TRP177 > 4 Å					
System	LinBWT		LinB32		LinB86	
	TIP3P	OPC	TIP3P	OPC*	TIP3P	OPC
1	95	99	12	1	23	5
2	97	97	24	61	4	6
3	98	98	24	2	32	4
4			24	18	23	11
5			43	1	31	21
6			5	6	38	28
7			6	2	35	
8			28	1	3	
9			20	1	41	
10				1		
11				1		
12				25		
13				2		
14				1		
15				1		
16				1		
17				9		
18				3		

The simulations chosen to represent open and closed states for further investigation are highlighted in yellow and red, respectively. The simulations were performed in batches of three replicates, until the two suitable representatives were found, with the exception of LinB32-OPC, in which after three such batches (3x3 simulations), additional 9 simulations were executed. * In case of LinB32-OPC, the simulations representing the closed state were selected from among the most closed ones, by also minimizing the deviation in the investigated distances.

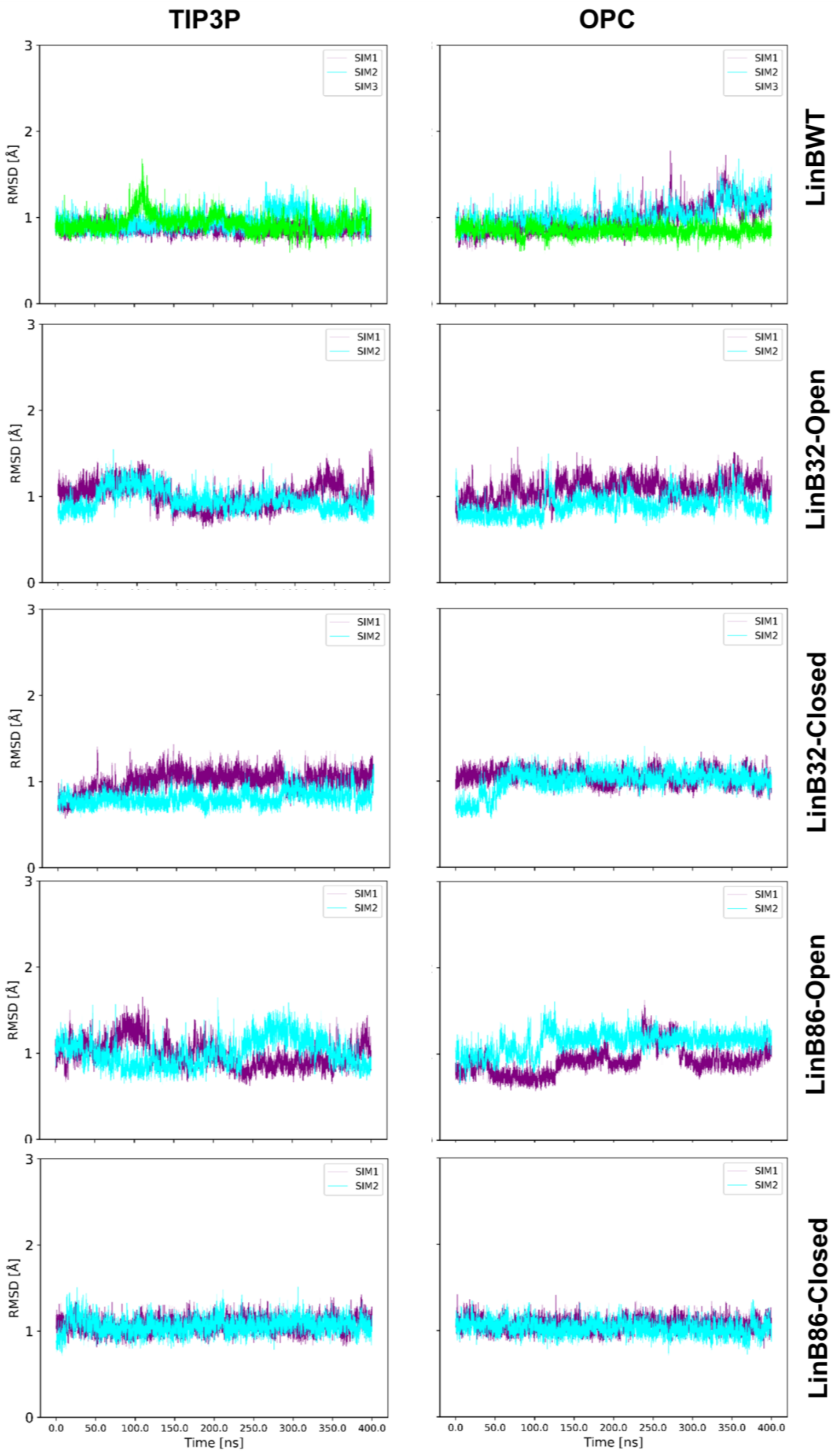

Figure S1. Time evolution of backbone RMSD in production phase investigated simulations in TIP3P and OPC water models.

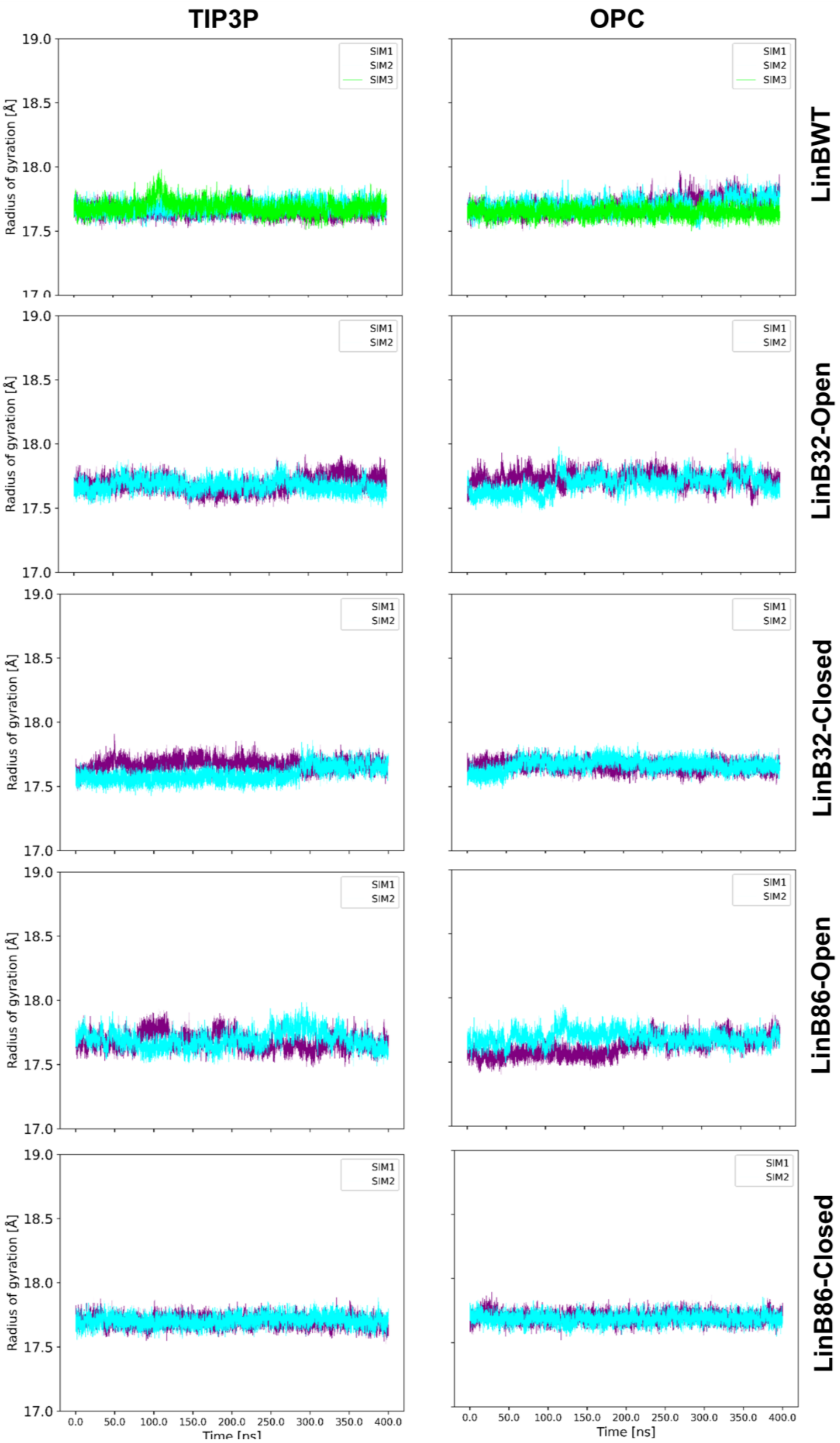

Figure S2. Time evolution of backbone radius of gyration in production phase of investigated simulations in TIP3P and OPC water models.

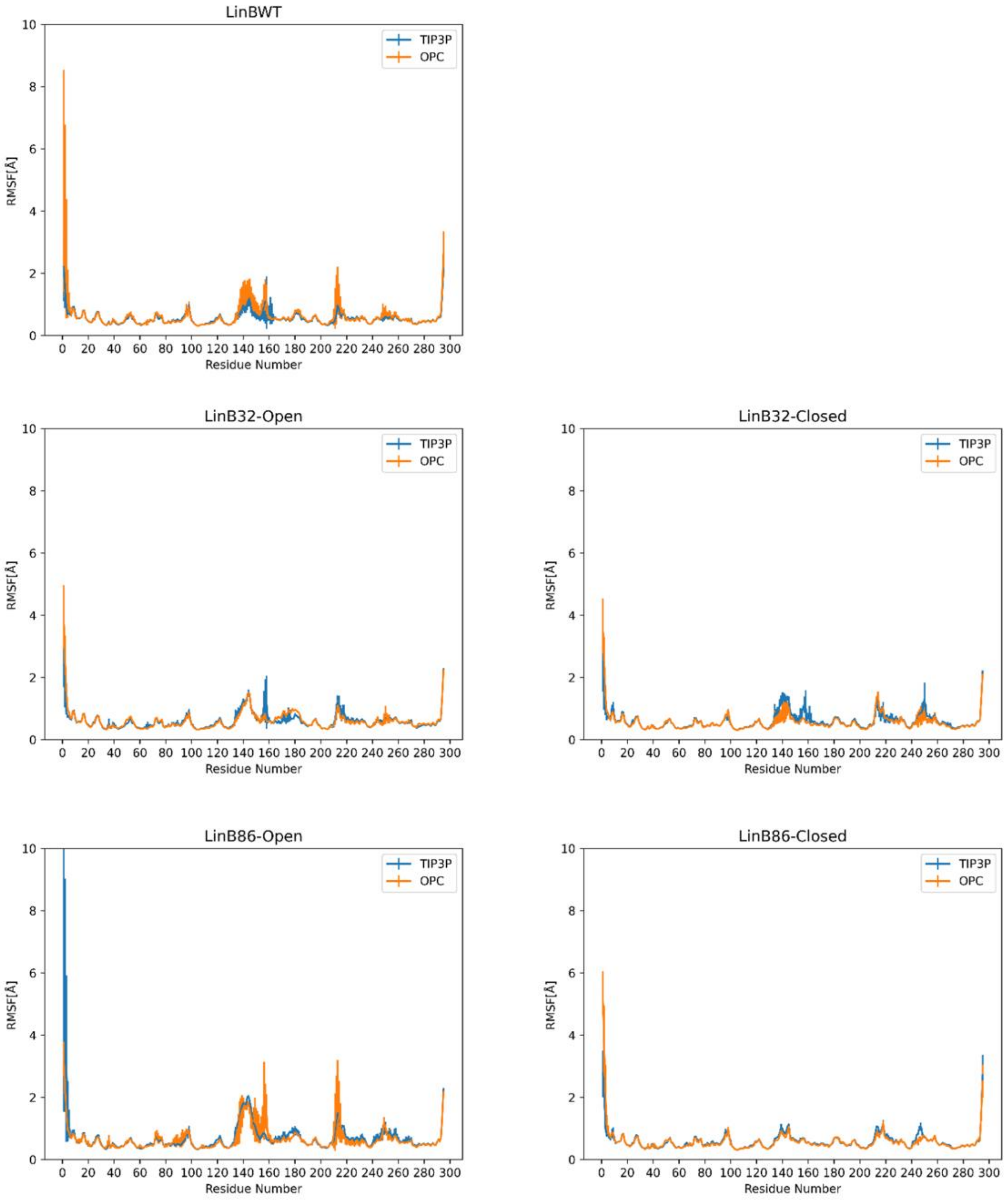

Figure S3. Average backbone RMSF of residues in production phase investigated simulations in TIP3P and OPC water models.

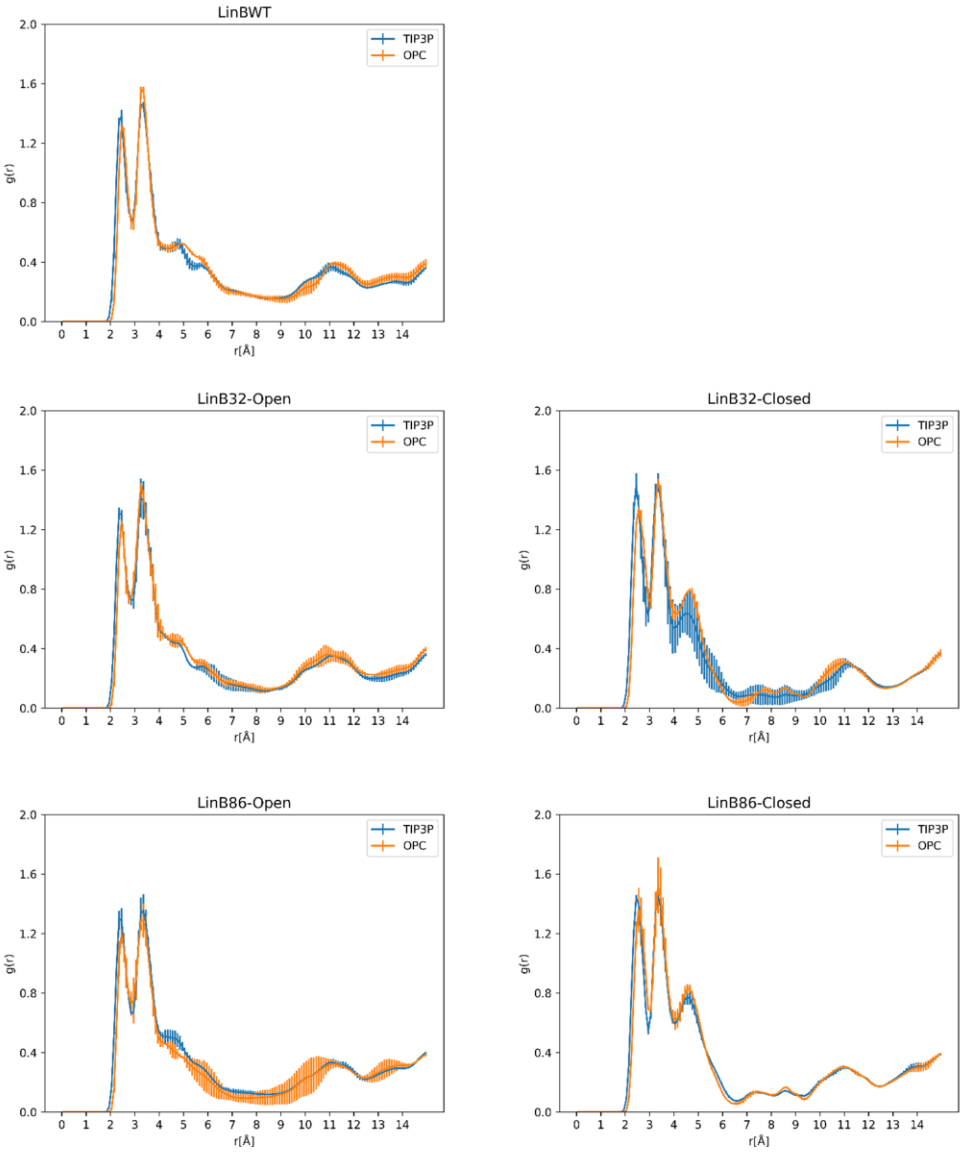

Figure S4. Average radial distribution function of water molecules around CG atom of Asp108 in investigated simulations in TIP3P and OPC water models.

Table S2. Results of number of waters in the first and second solvation for active site residues and bottleneck residues of P1 tunnel, including the results of two sample t-test.

System	Region	Solvation Shell	TIP3P		OPC		t-statistic	p-value
			Mean	SD	Mean	SD		
**LinBWT**	Active Site	First	6.14	1.21	6.55	1.28	0.2328	0.8274
		Second	10.93	1.78	11.34	2.20	0.1449	0.8918
	Bottleneck	First	19.31	2.69	20.35	3.10	0.2534	0.8125
		Second	38.76	4.06	40.35	5.14	0.2427	0.8201
**LinB32**	Active Site	First	6.20	1.76	6.99	1.63	0.3293	0.7531
		Second	10.31	2.74	11.74	2.79	0.3657	0.7271
	Bottleneck	First	17.28	4.30	18.21	4.58	0.1480	0.8872
		Second	33.81	6.62	35.83	6.84	0.2122	0.8390
**LinB86**	Active Site	First	6.96	1.63	6.94	1.60	0.0088	0.9933
		Second	11.21	2.35	11.02	2.29	0.0579	0.9957
	Bottleneck	First	17.47	3.61	17.24	3.56	0.0448	0.9657
		Second	34.94	5.42	34.37	5.27	0.0754	0.9423

Table S3A. Mean and standard deviation (SD) values for the Tunnel Detection - Absolute Value (Frames), Average Bottleneck Radius (Avg_BR), Max Bottleneck Radius (Max_BR) and Length (Len) for LinB systems and the two water models.

Tunnel	Frames	SD	Avg_BR *(Å)*	SD	Max_BR *(Å)*	SD	Len *(Å)*	SD	Model	System
**p1**	19929.000	24.880	1.797	0.045	2.588	0.048	10.401	0.440	OPC	LinBWT
**p2**	1856.000	3214.686	0.791	-	1.62	-	21.780	-	OPC	LinBWT
**p3**	5006.000	1369.202	0.787	0.029	1.627	0.358	20.561	1.300	OPC	LinBWT
**ST**	1525.667	1422.303	0.730	0.006	1.010	0.134	35.998	2.889	OPC	LinBWT
**p1**	15410.667	3384.586	1.794	0.037	2.489	0.079	11.091	0.089	TIP3P	LinBWT
**p2**	3252.667	1269.896	0.792	0.040	1.579	0.432	22.845	0.790	TIP3P	LinBWT
**p3**	5746.667	612.670	0.759	0.003	1.656	0.065	20.865	0.080	TIP3P	LinBWT
**ST**	2997.000	544.025	0.734	0.003	1.030	0.019	33.516	0.506	TIP3P	LinBWT
**p1**	16192.250	3263.929	1.198	0.447	2.051	0.446	12.710	1.376	OPC	LinB32
**p2**	10780.500	6661.317	1.051	0.165	1.983	0.132	19.948	1.586	OPC	LinB32
**p3**	2620.250	3464.777	0.768	0.026	1.219	0.250	19.356	0.708	OPC	LinB32
**ST**	2961.500	2495.702	0.742	0.008	1.069	0.060	30.910	0.395	OPC	LinB32
**p1**	17342.000	2906.874	1.240	0.324	2.294	0.173	12.908	0.568	TIP3P	LinB32
**p2**	10894.500	5286.020	0.947	0.161	1.905	0.087	20.053	1.271	TIP3P	LinB32
**p3**	5389.500	1139.447	0.776	0.021	1.369	0.115	19.528	1.153	TIP3P	LinB32
**ST**	3066.750	1298.469	0.734	0.008	0.999	0.061	31.731	2.185	TIP3P	LinB32
**p1**	14784.000	3342.503	1.018	0.265	1.937	0.470	13.206	1.178	OPC	LinB86
**p2**	12679.750	1845.948	1.055	0.130	1.984	0.083	19.411	0.588	OPC	LinB86
**p3**	11834.500	3261.131	0.867	0.060	1.690	0.277	16.158	0.286	OPC	LinB86
**ST**	1435.000	1033.531	0.728	0.004	1.020	0.160	34.093	1.155	OPC	LinB86
**p1**	15242.500	2775.916	1.132	0.317	2.281	0.275	13.086	1.208	TIP3P	LinB86
**p2**	8716.750	2963.842	1.001	0.065	1.978	0.142	19.911	1.043	TIP3P	LinB86
**p3**	13637.750	951.736	0.871	0.040	1.890	0.157	16.178	0.109	TIP3P	LinB86
**ST**	2972.000	525.176	0.736	0.006	1.085	0.119	32.670	0.988	TIP3P	LinB86

Table S3B. Results of two sample t-test performed for data represented in Figure 2 for LinBWT.

Tunnel	P1		P2		P3		ST	
Metric	**t-statistic**	**p-value**	**t-statistic**	**p-value**	**t-statistic**	**p-value**	**t-statistic**	**p-value**
**Avg_Frames**	2.3122	0.0818	-0.6999	0.5226	-0.8552	0.4406	-1.6735	0.1695
**Avg_BR**	0.0694	0.9480	NA	NA	1.6309	0.1782	0.9286	0.4216
**Max_BR**	1.8512	0.1378	NA	NA	-0.1395	0.8958	1.2173	0.3105
**Avg_Length**	-2.6600	0.0564	NA	NA	-0.4046	0.7065	0.0668	0.9509

Table S3C. Results of two sample t-test performed for data represented in Figure 2 for LinB32.

Tunnel	P1		P2		P3		ST	
Metric	**t-statistic**	**p-value**	**t-statistic**	**p-value**	**t-statistic**	**p-value**	**t-statistic**	**p-value**
**Avg_Frames**	-0.5261	0.6177	-0.0268	0.9795	-1.5185	0.1797	-0.0748	0.9428
**Avg_BR**	-0.1513	0.8847	0.9023	0.4017	-0.4351	0.6816	-0.3948	0.7131
**Max_BR**	-1.0163	0.3487	0.9889	0.3609	-1.0751	0.3314	-0.2517	0.8137
**Avg_Length**	-0.2663	0.7989	-0.1028	0.9214	-0.2246	0.8312	-0.2639	0.8049

Table S3D. Results of two sample t-test performed for data represented in Figure 2 for LinB86.

Tunnel	P1		P2		P3		ST	
Metric	**t-statistic**	**p-value**	**t-statistic**	**p-value**	**t-statistic**	**p-value**	**t-statistic**	**p-value**
**Avg_Frames**	-0.2111	0.8398	2.2700	0.0637	-1.0616	0.3293	-2.6516	0.0379
**Avg_BR**	-0.5558	0.5985	0.7585	0.4769	-0.0907	0.9307	0.3021	0.7747
**Max_BR**	-1.2661	0.2524	0.0757	0.9421	-1.2546	0.2563	-0.4591	0.6654
**Avg_Length**	0.1422	0.8916	-0.8365	0.4349	-0.1291	0.9015	-1.7157	0.1469

**Welch t-test performed revealed statistically significantt differences for the Avg_BR of LinBWT and LinB32 in the TIP3P model (p = 0.0428), while the OPC comparison was not significant (p = 0.0762). For LinBWT and LinB86, both OPC (p = 0.0104) and TIP3P (p = 0.0256) showed statistically significant differences for Avg_BR.*

Table S4A. Comparison of characteristcis of all tunnel clusters identified in TIP3P and OPC water model in LinBWT.

Cluster	No. of Simulations		Total Frames		Average Radii Bottleneck (Å)		Maximum Bottleneck Radii (Å)		Average Length (Å)	
**CID**	OPC	TIP3P	OPC	TIP3P	OPC	TIP3P	OPC	TIP3P	OPC	TIP3P
1	1	1	59787	46232	1.797 ± 0.224	1.796 ± 0.199	2.642	2.552	10.401 ± 1.655	11.079 ± 1.132
2	2	2	8080	12805	0.818 ± 0.150	1.791 ± 0.194	2.17	2.378	17.759 ± 3.439	11.368 ± 1.135
3	1	3	5568	9758	0.791 ± 0.091	0.803 ± 0.128	1.62	1.839	21.780 ± 2.305	22.644 ± 2.442
4	3	3	15018	17240	0.787 ± 0.107	0.759 ± 0.063	1.924	1.712	20.668 ± 3.497	20.864 ± 2.503
5	3	3	7423	14224	0.766 ± 0.072	0.775 ± 0.076	1.341	1.361	23.755 ± 3.175	23.875 ± 3.429
6	3	3	21329	26140	0.758 ± 0.054	0.763 ± 0.058	1.16	1.205	26.266 ± 3.700	26.075 ± 3.362
7	3	2	24739	5504	0.842 ± 0.147	0.800 ± 0.106	1.823	1.676	19.125 ± 2.818	19.177 ± 2.738
8	2	3	6339	11458	0.962 ± 0.252	0.828 ± 0.143	2.173	2.046	21.649 ± 2.576	20.395 ± 2.603
9	-	-	-	-	-	-	-	-	-	-
10	-	-	-	-	-	-	-	-	-	-
11	2	3	4577	8991	0.731 ± 0.037	0.734 ± 0.037	1.105	1.043	35.528 ± 7.719	33.457 ± 6.943
12	3	3	4405	5835	0.730 ± 0.034	0.731 ± 0.034	1.365	1.081	30.687 ± 7.595	29.282 ± 6.503
13	-	1	-	1660	-	0.740 ± 0.042	-	0.947	-	32.983 ± 4.853
14	-	1	-	1128	-	0.759 ± 0.061	-	1.112	-	27.009 ± 3.300
15	1	2	3963	2770	0.799 ± 0.126	0.778 ± 0.097	1.797	1.692	18.534 ± 3.092	19.225 ± 3.178
16	1	1	1539	6788	0.803 ± 0.096	0.782 ± 0.088	1.26	1.265	31.352 ± 4.510	31.889 ± 4.992
17	-	3	-	5224	-	0.739 ± 0.038	-	1.04	-	31.667 ± 5.387
18	2	2	2681	3037	0.729 ± 0.030	0.729 ± 0.029	0.914	0.946	30.106 ± 3.796	31.131 ± 3.779
19	-	3	-	5881	-	0.740 ± 0.042	-	1.222	36.091 ± 5.925	-
20	-	1	-	1395	-	0.754 ± 0.052	-	1.12	--	21.029 ± 2.350
21	3	-	8527	-	0.735 ± 0.037	-	1.105	-	33.223 ± 6.178	-
22	-	2	-	2987	-	0.731 ± 0.031	-	0.946	-	48.677 ± 5.075
23	-	-	-	-	-	-	-	-	-	-
24	-	2	-	2608	-	0.723 ± 0.023	-	0.897	-	36.440 ± 6.085
25	-	-	-	-	-	-	-	-	-	-
26	-	-	-	-	-	-	-	-	-	-
27	-	-	-	-	-	-	-	-	-	-
28	-	-	-	-	-	-	-	-	-	-
29	-	2	-	2873	-	0.728 ± 0.029	-	0.951	-	47.479 ± 6.664
30	-	1	-	1095	-	0.725 ± 0.025	-	0.856	-	64.230 ± 5.994
31	1	-	1678	-	0.752 ± 0.062	-	1.258		25.822 ± 4.101	-
32	-	-	-	-	-	-	-	-	-	-
33	-	-	-	-	-	-	-	-	-	-
34	-	-	-	-	-	-	-	-	-	-
35	1	-	1066	-	0.726 ± 0.025	-	0.902	-	36.695 ± 5.371	-
36	-	-	-	-	-	-	-	-	-	-

Table S4B. Comparison of characteristics of all tunnel clusters identified in TIP3P and OPC water model in LinB32.

Cluster	No. of Simulations		Total Frames		Average Bottleneck Radii (Å)		Maximum Bottleneck Radii (Å)		Average Length (Å)	
**CID**	OPC	TIP3P	OPC	TIP3P	OPC	TIP3P	OPC	TIP3P	OPC	TIP3P
1	4	4	64769	69368	1.260 ± 0.461	1.276 ± 0.398	2.482	2.448	12.514 ± 2.465	12.849 ± 1.995
2	4	4	52566	60032	1.261 ± 0.436	1.239 ± 0.375	2.42	2.558	13.950 ± 2.789	14.052 ± 2.627
3	4	4	43122	43578	1.111 ± 0.272	1.005 ± 0.273	2.108	1.996	19.226 ± 2.153	19.670 ± 2.873
4	3	4	10481	21558	0.787 ± 0.090	0.774 ± 0.081	1.503	1.533	18.990 ± 3.478	19.640 ± 3.127
5	4	4	49873	34884	0.860 ± 0.118	0.849 ± 0.134	1.555	1.763	22.296 ± 2.709	22.391 ± 3.022
6	4	4	46562	35277	0.788 ± 0.077	0.779 ± 0.073	1.284	1.296	26.210 ± 3.910	25.880 ± 4.041
7	3	3	17055	26932	0.813 ± 0.119	0.869 ± 0.147	1.736	1.82	16.749 ± 2.462	16.667 ± 2.401
8	2	-	17240	-	1.041 ± 0.287	-	2.251	-	17.812 ± 3.611	-
9	2	1	27017	15124	0.857 ± 0.136	0.860 ± 0.130	1.559	1.567	21.816 ± 2.866	23.945 ± 3.467
10	3	1	16810	7826	0.761 ± 0.062	0.770 ± 0.071	1.288	1.635	27.598 ± 5.072	27.457 ± 4.806
11	3	4	11846	12267	0.745 ± 0.048	0.737 ± 0.041	1.113	1.04	30.965 ± 6.797	31.142 ± 7.088
12	2	4	5620	8065	0.745 ± 0.048	0.734 ± 0.038	1.163	1.09	27.842 ± 5.201	28.278 ± 6.424
13	2	3	11657	8002	0.842 ± 0.118	0.792 ± 0.097	1.537	1.471	28.050 ± 3.009	30.537 ± 5.156
14	2	1	5734	4920	0.770 ± 0.068	0.772 ± 0.069	1.141	1.239	29.250 ± 5.037	28.495 ± 4.726
15	2	1	2956	1975	0.785 ± 0.106	0.779 ± 0.093	1.885	1.758	18.024 ± 3.286	18.980 ± 3.827
16	2	3	8428	10811	0.791 ± 0.091	0.785 ± 0.088	1.574	1.337	30.635 ± 4.591	30.957 ± 5.487
17	3	3	7067	4653	0.740 ± 0.039	0.740 ± 0.039	1.007	0.984	31.539 ± 5.761	30.839 ± 5.511
18	2	3	4032	4785	0.731 ± 0.031	0.729 ± 0.030	0.923	0.939	30.871 ± 4.463	30.214 ± 3.781
19	3	2	4216	5376	0.744 ± 0.046	0.744 ± 0.045	1.102	1.143	35.462 ± 6.302	36.517 ± 5.419
20	3	2	8950	2533	0.751 ± 0.059	0.750 ± 0.051	1.66	1.132	20.571 ± 2.916	20.284 ± 2.677
21	-	1	-	1989	-	0.736 ± 0.035	-	0.967	-	31.833 ± 5.272
22	3	3	4913	5316	0.727 ± 0.029	0.731 ± 0.033	0.943	0.96	45.349 ± 5.752	46.348 ± 5.688
23	2	-	7083	-	0.830 ± 0.139	-	2.013	-	20.425 ± 5.095	-
24	2	2	5108	2868	0.730 ± 0.030	0.724 ± 0.025	0.935	0.918	33.762 ± 5.197	33.982 ± 5.871
25	1	1	1353	1123	0.745 ± 0.040	0.741 ± 0.037	0.94	0.921	36.151 ± 4.611	35.396 ± 4.475
26	-	-	-	-	-	-	-	-	-	-
27	-	-	-	-	-	-	-	-	-	-
28	2	1	2655	1347	0.731 ± 0.033	0.732 ± 0.033	0.997	0.983	34.946 ± 5.789	33.453 ± 4.995
29	2	2	3453	2219	0.732 ± 0.031	0.728 ± 0.029	0.901	0.912	46.285 ± 5.978	47.132 ± 6.949
30	1	2	1083	3423	0.723 ± 0.024	0.723 ± 0.023	0.921	0.893	55.470 ± 5.788	58.507 ± 7.628
31	-	1	-	1274	-	0.746 ± 0.053	-	1.142	-	24.360 ± 3.696
32	2	1	2748	1461	0.731 ± 0.031	0.730 ± 0.030	0.909	0.899	30.319 ± 5.121	29.555 ± 4.019
33	-	1	-	1865	-	0.738 ± 0.038	-	0.959	-	31.044 ± 5.375
34	-	2	-	2433	-	0.727 ± 0.025	-	0.872	-	35.695 ± 5.172
35	2	-	4029	-	0.740 ± 0.043	-	1.041	-	29.540 ± 6.371	
36	-	-	-	-	-	-	-	-	-	-

Table S4C. Comparison of characteristics of all tunnel clusters identified in TIP3P and OPC water model in LinB86.

Cluster	No. of Simulations		Total Frames		Average Bottleneck Radii (Å)		Maximum Bottleneck Radii (Å)		Average Length (Å)	
CID	OPC	TIP3P	OPC	TIP3P	OPC	TIP3P	OPC	TIP3P	OPC	TIP3P
1	4	4	59136	60970	1.060 ± 0.353	1.174 ± 0.413	2.4	2.566	13.014 ± 2.438	12.926 ± 2.251
2	4	4	48680	59330	0.904 ± 0.264	1.025 ± 0.335	2.292	2.393	15.158 ± 2.834	14.699 ± 2.933
3	4	4	50719	34867	1.067 ± 0.275	1.012 ± 0.247	2.074	2.114	19.411 ± 2.217	19.705 ± 2.046
4	4	4	47338	54551	0.862 ± 0.154	0.873 ± 0.163	1.938	2.09	16.118 ± 2.323	16.175 ± 2.208
5	4	4	44159	54282	0.880 ± 0.131	0.884 ± 0.133	1.569	1.655	23.610 ± 3.128	23.370 ± 3.536
6	4	4	33699	48859	0.794 ± 0.082	0.810 ± 0.092	1.239	1.334	28.636 ± 4.673	27.373 ± 4.539
7	4	3	13513	17568	0.873 ± 0.213	0.849 ± 0.140	1.937	1.78	16.661 ± 2.939	17.703 ± 2.842
8	3	4	9548	29945	0.886 ± 0.176	1.074 ± 0.304	2.03	2.34	17.986 ± 2.459	16.847 ± 3.086
9	2	3	29757	37342	0.907 ± 0.139	0.963 ± 0.168	1.629	1.997	21.823 ± 2.861	22.366 ± 2.966
10	2	4	22908	33024	0.778 ± 0.073	0.777 ± 0.075	1.254	1.296	27.789 ± 4.290	28.829 ± 5.231
11	3	4	5740	11888	0.729 ± 0.033	0.736 ± 0.042	1.204	1.26	34.029 ± 7.977	32.799 ± 7.653
12	2	4	2894	8777	0.731 ± 0.033	0.736 ± 0.042	0.984	1.211	29.478 ± 7.265	28.642 ± 6.322
13	2	1	11319	1179	0.980 ± 0.148	0.789 ± 0.094	1.579	1.239	23.260 ± 2.735	31.159 ± 5.691
14	2	4	4823	16227	0.756 ± 0.053	0.769 ± 0.069	1.095	1.27	32.092 ± 4.545	31.412 ± 5.912
15	2	1	3762	1105	0.808 ± 0.120	0.784 ± 0.094	1.502	1.702	16.653 ± 4.216	19.359 ± 3.596
16	1	-	2728	-	0.760 ± 0.066	-	1.54	-	32.231 ± 6.720	-
17	1	3	1465	6667	0.750 ± 0.046	0.741 ± 0.039	0.986	1.083	29.756 ± 6.165	30.744 ± 5.588
18	2	3	2940	4730	0.730 ± 0.031	0.733 ± 0.034	0.975	1.01	29.811 ± 4.105	30.019 ± 3.825
19	1	2	2737	3488	0.738 ± 0.040	0.745 ± 0.045	0.96	1.008	35.623 ± 4.562	36.571 ± 5.363
20	-	-	-	-	-	-	-	-	-	-
21	2	1	2674	2508	0.740 ± 0.041	0.742 ± 0.040	0.996	0.951	30.315 ± 5.578	32.163 ± 5.813
22	3	2	4227	3178	0.731 ± 0.033	0.733 ± 0.034	0.971	0.936	44.443 ± 4.528	45.809 ± 4.943
23	-	1	-	1266	-	0.836 ± 0.141	-	1.929	-	19.531 ± 4.279
24	-	2	-	3366	-	0.727 ± 0.027	-	0.953	-	35.371 ± 5.690
25	2	3	2308	3581	0.739 ± 0.037	0.742 ± 0.040	0.978	1.003	36.630 ± 5.781	35.261 ± 4.638
26	2	-	7501	-	0.762 ± 0.064	-	1.223	-	25.013 ± 5.051	-
27	1	-	4659	-	0.806 ± 0.104	-	1.69	-	16.325 ± 1.598	-
28	1	3	1047	3759	0.730 ± 0.033	0.728 ± 0.030	0.969	0.953	33.766 ± 5.353	36.014 ± 6.423
29	1	1	1101	1761	0.740 ± 0.040	0.732 ± 0.032	0.953	0.927	43.133 ± 5.116	45.637 ± 5.621
30	2	4	2898	5600	0.723 ± 0.024	0.724 ± 0.026	0.91	0.93	54.682 ± 5.257	58.203 ± 7.175
31	1	-	1391	-	0.765 ± 0.071	-	1.158	-	21.141 ± 3.602	-
32	-	1	-	1000	-	0.729 ± 0.028	-	0.929	-	31.945 ± 3.987
33	1	1	3052	1064	0.737 ± 0.039	0.747 ± 0.051	1.034	1.193	30.330 ± 6.032	31.877 ± 5.992
34	-	-	-	-	-	-	-	-	-	-
35	-	1	-	1010	-	0.727 ± 0.027	-	0.863	-	34.756 ± 5.796
36	-	-	-	-	-	-	-	-	-	-

Table S5. Results of two sample t-test performed for data represented in Figure 3.

Enzyme	LinBWT		*LinB32*		*LinB86*	
Bin Center [Å]	**t-statistic**	**p-value**	**t-statistic**	**p-value**	**t-statistic**	**p-value**
**0.70**	-1.3868	0.2378	-0.3743	0.7211	-0.3498	0.7384
**0.80**	-1.4980	0.2085	-0.3280	0.7540	-0.4019	0.7017
**0.90**	-1.4130	0.2305	-0.1110	0.9152	-0.6608	0.5333
**1.00**	-1.4104	0.2312	0.3089	0.7679	-0.4336	0.6798
**1.10**	-1.5152	0.2043	1.5031	0.1835	-0.1671	0.8728
**1.20**	-0.8944	0.4217	2.4419	0.0503	0.0280	0.9786
**1.30**	-0.9993	0.3742	1.6144	0.1576	0.0894	0.9316
**1.40**	-1.1590	0.3109	1.1939	0.2776	0.1518	0.8844
**1.50**	-0.9991	0.3743	0.7589	0.4767	0.1980	0.8496
**1.60**	-1.1575	0.3115	0.3962	0.7057	0.3898	0.7102
**1.70**	-1.3545	0.2470	0.1118	0.9146	0.5248	0.6185
**1.80**	-1.4336	0.2250	-0.1609	0.8774	0.7840	0.4629
**1.90**	-1.3633	0.2445	-0.4236	0.6866	1.0395	0.3386
**2.00**	-1.3166	0.2583	-0.5900	0.5767	1.1446	0.2960
**2.10**	-1.8801	0.1333	-0.7543	0.4792	1.2267	0.2659
**2.20**	-1.8924	0.1314	-0.9520	0.3779	1.3851	0.2153
**2.30**	-1.7320	0.1583	-1.0410	0.3380	1.5987	0.1610
**2.40**	-1.6594	0.1724	-1.0912	0.3170	1.4280	0.2032
**2.50**	-2.0642	0.1079	-1.0000	0.3559	1.0000	0.3559

Table S6. Results of two sample t-test performed for data represented in Figure 4

Enzyme	*LinBWT*		*LinB32*		*LinB86*	
Bin Center [Å]	***t-statistic***	***p-value***	***t-statistic***	***p-value***	***t-statistic***	***p-value***
**0.02**	-3.5402	0.0240	-1.0397	0.3386	-1.3760	0.2180
**0.05**	-2.4417	0.0711	2.3849	0.0544	1.6077	0.1590
**0.10**	-0.3786	0.7242	1.1348	0.2998	1.0981	0.3142
**0.15**	1.9792	0.1189	0.9270	0.3897	1.9546	0.0984
**0.20**	0.1347	0.8994	0.1938	0.8527	1.0028	0.3547
**0.25**	1.1355	0.3196	0.3500	0.7383	0.4669	0.6570
**0.30**	0.9456	0.3979	-0.1196	0.9087	0.9146	0.3957
**0.35**	3.2082	0.0326	-0.5130	0.6263	0.2465	0.8135
**0.40**	1.5285	0.2011	-0.4606	0.6613	0.6680	0.5290
**0.45**	2.0862	0.1053	-0.2981	0.7757	0.2736	0.7936
**0.50**	0.6917	0.5272	-0.6641	0.5313	0.3453	0.7416
**0.55**	2.0461	0.1102	-0.6795	0.5221	0.2614	0.8025
**0.60**	1.4816	0.2126	-0.4891	0.6422	0.4847	0.6451
**0.65**	0.9909	0.3778	-1.1438	0.2963	-0.5522	0.6008
**0.70**	2.8016	0.0487	-0.5008	0.6343	-0.4713	0.6541
**0.75**	1.3753	0.2410	-1.2426	0.2604	0.3920	0.7086
**0.80**	1.1548	0.3125	-1.3377	0.2295	1.2125	0.2709
**0.85**	2.6415	0.0575	-0.4570	0.6637	0.3813	0.7161
**0.90**	2.0207	0.1134	-1.0705	0.3256	-0.1675	0.8725
**0.95**	1.1436	0.3166	-1.1600	0.2901	-1.2732	0.2501
**1.00**	1.9181	0.1276	-1.2436	0.2600	-0.0539	0.9587
**2.00**	-0.1836	0.8632	-1.1945	0.2774	0.6951	0.5130
**4.00**	-0.8623	0.4372	-1.0945	0.3157	1.5445	0.1734
**6.00**	-1.5519	0.1956	-1.0000	0.3559	NA	NA
**8.00**	0.0534	0.9600	-1.0000	0.3559	NA	NA

References

Brezovsky, J., Babkova, P., Degtjarik, O., Fortova, A., Gora, A., Iermak, I., Rezacova, P., Dvorak, P., Smatanova, I. K., Prokop, Z., Chaloupkova, R., & Damborsky, J. (2016). Engineering a de Novo Transport Tunnel. *ACS Catalysis*, *6*(11), 7597–7610. https://doi.org/10.1021/acscatal.6b02081

Kokkonen, P., Sykora, J., Prokop, Z., Ghose, A., Bednar, D., Amaro, M., Beerens, K., Bidmanova, S., Slanska, M., Brezovsky, J., Damborsky, J., & Hof, M. (2018). Molecular Gating of an Engineered Enzyme Captured in Real Time. *Journal of the American Chemical Society*, *140*(51), 17999–18008. https://doi.org/10.1021/jacs.8b09848
